# Identification of High Serum Apolipoprotein A1 as a Favorable Prognostic Indicator in Patients with Multiple Myeloma

**DOI:** 10.7150/jca.31357

**Published:** 2019-08-27

**Authors:** Lifan Liang, Jing Li, Hangcheng Fu, Xinyang Liu, Peng Liu

**Affiliations:** 1 Department of Hematology, Zhongshan Hospital, Fudan University, Shanghai, China; 2 Fudan University Shanghai Cancer Center, Shanghai, China

**Keywords:** multiple myeloma, lipid profile, apolipoprotein A1, prognosis, prognostic model

## Abstract

This study is to explore the prognostic significance of serum lipid profiles in patients with multiple myeloma (MM). The study retrospectively enrolled 307 MM patients in Zhongshan Hospital, Shanghai, China, from 2007 to 2016. We evaluated the prognostic significance of the pre-diagnostic serum lipid profile [cholesterol, triglyceride, low-density lipoprotein (LDL), high-density lipoprotein (HDL), Apolipoprotein A1 (Apo A1) and Apolipoprotein B (Apo B)]. Prognostic factors identified through univariate and multivariate analysis were used to construct a new model based on Lasso Cox regression. Results indicated that lipid levels showed significant difference between ISS stages: Apo A1, Apo B, Cholesterol and LDL levels were lower in late ISS stage. However, only Apo A1 showed statistically significance in overall survival (OS), progression free survival (PFS) and cause specific survival (CSS) (*P*=0.038, *P*=0.028, *P*=0.011) in univariate Cox regression. Patients with higher Apo A1 displayed longer OS (median OS, 67 months *vs.* 30 months; *P*<0.001). Also, Apo A1 was revealed to be an independent prognostic indicator through multivariate analysis. Combining the Apo A1 level, Zhongshan Score model was constructed with Lasso regression for prognosis prediction. This model exhibited higher accuracy than International Staging System (ISS) and Durie and Salmon (DS) system. In conclusion, among all the serum lipid profiles, serum Apo A1 is a powerful prognostic indicator for patients with MM.

## Introduction

Multiple myeloma (MM) is a plasma cell disease with great heterogeneity, with the survival time ranging from several months to more than 10 years 1. Despite the advent of more effective therapies and improvements in supportive care, which has led to an increased median survival to 6 years, this variety of prognosis still exists 2.

Several prognostic evaluation systems have been applied in clinical work. In 1975, Durie and Salmon (DS) system was introduced 3. However, the quantitation of lytic bone lesion in this system is observer-dependent, and thus decreased its precision. In 2005, the International Staging System (ISS) provided clinical practitioners with a simple risk stratification algorithm, and thus was widely used in clinical practicing 4. Revised ISS (R-ISS), combining the cytogenetic abnormality and serum lactate dehydrogenase (LDH) level with the ISS system, turned out to be more accurate in prediction 5. However, the R-ISS requires cytogenetic analysis, such as fluorescence in situ hybridization (FISH), which is a big burden for health care system in some underdeveloped area. Exploring new prognostic factors is still a necessity for MM management. With a deeper understanding of prognostic factors, we can identify high risk patient group, estimate treatment response, and provide tailored therapy.

Deregulation of lipid metabolism is one of the characteristics of cancer cell, and it is considered as a therapeutic target for cancer 6. Lipid metabolism have long been investigated in myeloma patients and the studies have shown that abnormal lipid level was seen in the patients with MM, compared with those in healthy controls^7^, ^8^. Moreover, statins, an HMG-CoA reductase inhibitor used in treating hyperlipidemia, were found to have anti-myeloma effect both *in vivo* and *in vitro* studies, probably by increasing the susceptibility of apoptosis in myeloma cells through multiple pathways 9-11. Several studies showed that statins were associated with reduced mortality in MM, and also they might increase sensitivity and decrease drug resistance to therapy in relapsed and refractory myeloma 11-14. The evidence of the correlation between lipid and myeloma inspired us to carry out a more detailed study in the lipid profile in myeloma patients.

Apolipoprotein A1 (Apo A1), an essential part in high density lipoprotein (HDL), was proved to have additional function besides lipid metabolism 15. A population-based prospective cohort study by Signe Borgquist et al. reported that circulating levels of apolipoprotein (Apo A1 and Apo B) were associated with overall cancer risk in men and with breast, lung and colorectal cancer in both genders. Moreover, Apo A1 level was found to be inversely associated with lung cancer risk 16. Other studies in solid tumors such as colon cancer, breast cancer, ovarian cancer, small cell lung cancer and nasopharyngeal cancer, also reported that higher Apo A1 level might be a potential factor to predict better outcome^17^-^20^. However, no study has ever focused on the connection between lipid profiles and MM prognosis. Given the significance of metabolism chaos in MM patients, investigating the prognosis value of lipid profiles might not only aid clinical decision but also help to have deeper recognition of tumorigenesis of MM. Therefore, this study is to evaluate the prognosis value of serum lipid profiles in patients with MM.

## Patients and Methods

### Patient Selection

This was a retrospective study, approved by the Clinical Research Ethics Committee of Fudan University affiliated Zhongshan Hospital. From 2007 to 2016, the recorded cases of diagnosed MM in Zhongshan Hospital were 361. Exclusion criteria were listed as following: (a). Patients diagnosed with Monoclonal gammopathy of undetermined significance, smoldering multiple myeloma, primary amyloidosis or POEMS syndrome and solitary extramedullary plasmacytoma; (b). Patients with a prior chemotherapy treatment in other clinical centers; (c). Patients died or lost follow-up within one month; (d). Unavailable lipid profile data. A total number of 307 patients were included for final analysis, all patients met the diagnostic criteria of MM 21. Ten patients were excluded for non-newly diagnosed MM with a prior treatment in other clinical centers. Twenty patients were excluded for absence of lipid profile. Twenty-three patients died or lost follow-up in the first month after diagnosis. One patient was diagnosed as solitary extramedullary plasmacytoma. Baseline data were collected after the date of diagnosis and before the initiation of treatment, including laboratory parameters such as monoclonal immunoglobulin isotypes and concentration, serum lipid profile, serum calcium, serum creatinine (SCr), serum β2-microglobulin (β2MG), serum lactate dehydrogenase (LDH), albumin, globulin and hemoglobin. Serum Apolipoprotein were assayed by immunoturbidimetry with Dyasys kit, and other lipid profiles such as cholesterol and triglyceride were assayed by colorimetry with Roche's kit. Blood sample for lipid analysis was taken after strict fasting for at least 6 hours. Due to the limitation of techniques in our hospital and the related medical health insurance policy during the time period in our data, only 96 patients had undertaken FISH analysis and 123 patients had the record of free light chain (FLC) level. Data of FISH and FLC was collected in our study but not analyzed due to the incompleteness of data. Other information such as age, gender, isotypes, DS stage, ISS stage, date of diagnosis, and date of progression, date of death or last follow-up, and treatment strategy were also collected.

The follow-up period ended in April 2017. The median follow-up time is 15 months (from 1 month to 102 months). At the time of analysis, 100 patients died, and 43 patients lost follow-up. The primary endpoint was OS, defined as the time from the date of diagnosis to the most recent follow-up or the date of death. The second end point was progression free survival (PFS) which is defined as the time from the date of diagnosis to the date of most recent follow-up or the date of disease progression per International Myeloma Working Group consensus criteria (see supplement document) 22. Cause specific survival (CSS) was defined as the time from the date of diagnosis to the most recent follow-up or the date of death caused by myeloma.

### Statistical Analysis

The One-way ANOVA and student t-test of lipid profiles in different ISS stage was performed using GraphPad Prism (version 5.01 for Windows, GraphPad Software, San Diego California USA, www.graphpad.com). The univariate and multivariate of Cox regression analysis, the correlation analysis and the Kaplan Meier survival curve were performed with MedCalc software (version 12.7.0.0 for Windows, Medcalc software, www.medcalc.org). Smooth HR, Lasso Cox regression and time-dependent Receiver operating characteristic (ROC) were performed by R software packages, version 3.4.1 (The R foundation for Statistical Computing, www.-rproject.org). The coefficients and partial likelihood deviance were calculated with “glmnet” package in R. We also assessed the prognostic accuracy of the new models by Harell concordance index (*C*-index) and Akaike information criterion (AIC), which was performed by Stata 12.0 (StataCorp, College Station, TX). Two-sided *P* values less than 0.05 was regarded as statistically significant.

## Results

### Serum Lipid Profile Analysis in Different ISS Stages

**Figure 1:**
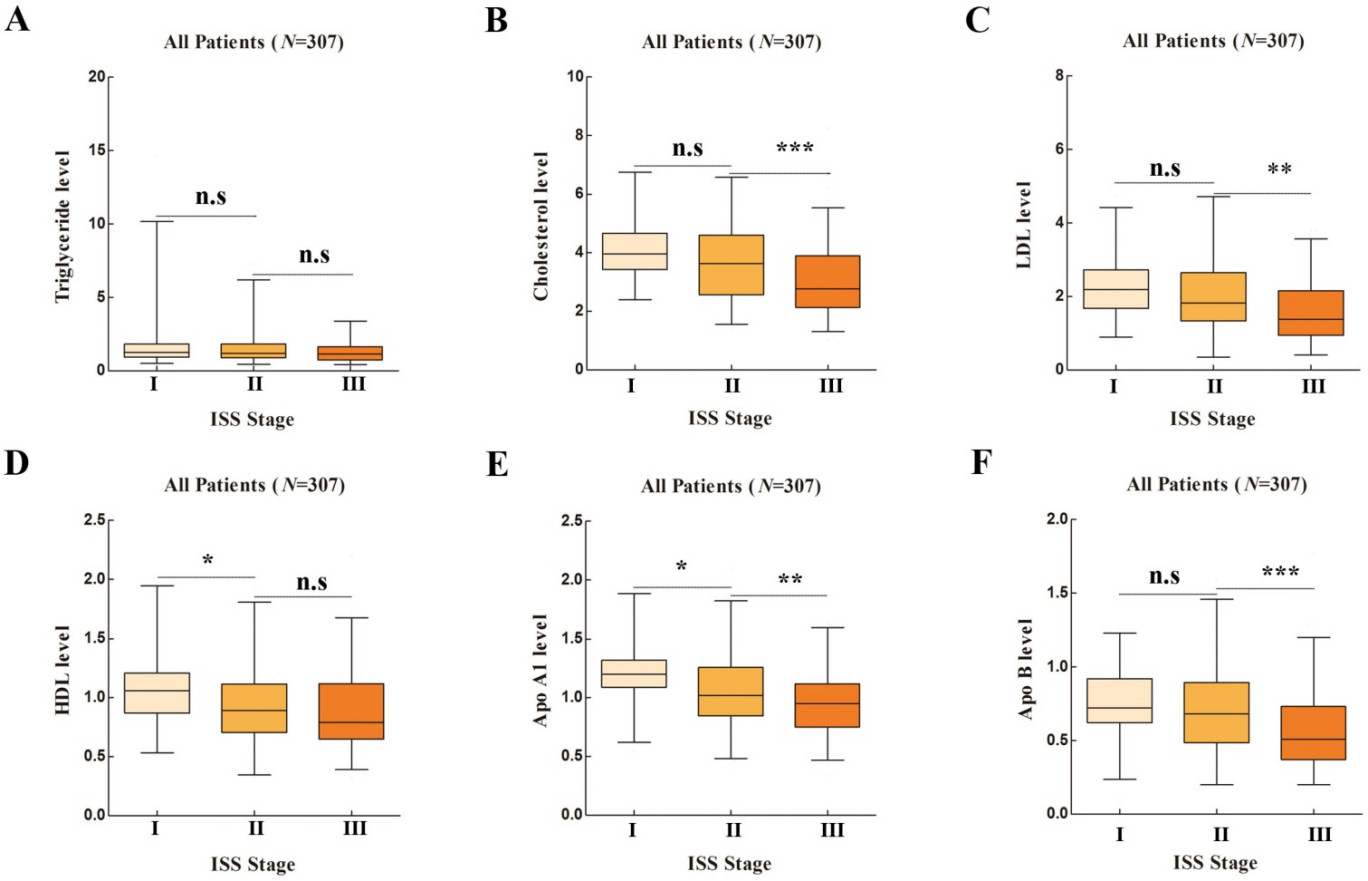
** Lipid Profiles in Different International Staging System (ISS) Stages in One-way ANOVA Analysis.** [A], Apolipoprotein A1 (Apo A1); [B], Apolipoprotein B (Apo B); [C], Cholesterol; [D], High density lipoprotein (HDL); [E], Low density lipoprotein (LDL); [F], Triglyceride.

Baseline information was listed in Table S1. The correlation between serum lipid profiles and ISS stages were compared in Figure 1. Serum Cholesterol, LDL, HDL, Apo A1, Apo B1 showed significance in ANOVA analysis (*P*<0.001, *P*<0.001, *P*=0.002, *P*<0.001 and *P*<0.001, respectively). Serum Apo A1 showed both significant difference in stage I vs. stage II and stage II vs. stage III (*P*=0.007 and *P*=0.002, respectively).

### Apo A1 Identified as an Independent Prognostic Factor through Survival Analysis

**Figure 2:**
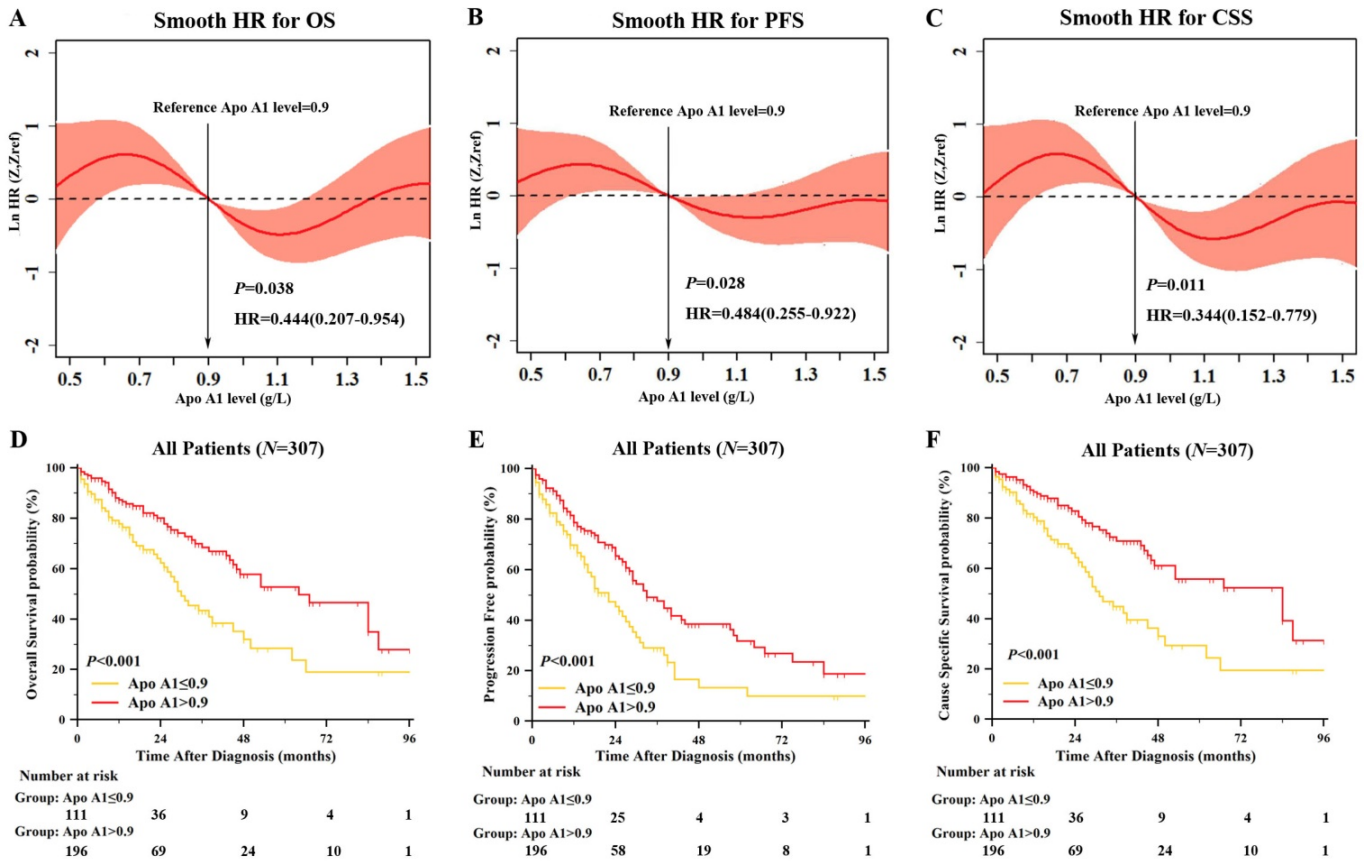
** Smooth HR Analysis and Kaplan Meier Survival Analysis of Apolipoprotein A1 (Apo A1).** Smooth HR analysis of Apo A1 in overall survival (OS) [A], progression free survival (PFS) [B], cause specific survival (CSS) [C] showed that HR was decreasing as Apo A1 was increasing; Kaplan Meier survival analysis between group of Apo A1 ≤0.9g/L and Apo A1 >0.9 g/L showed significant difference in OS [D], PFS [E] and CSS [F].

When further exploring the prognostic role of lipid profile, only continuous Apo A1 level was significant correlated to OS in univariate Cox regression (hazard ratio (HR): 0.444, 95% confidence interval (95% CI): 0.207-0.954,* P*=0.038). Apo A1 level was also significantly correlated with PFS and CSS (HR: 0.484 and 0.344, 95% CI: 0.255-0.922 and 0.152-0.779,* P*=0.028 and* P*=0.011, respectively). To evaluate the prognostic value of Apo A1 as a linear variable, we performed smooth HR analysis of OS, PFS and CSS (Figure 2A-2C). We chose 0.9g/L as the cutoff point to dichotomize Apo A1 as shown in Figure 2. As the results exhibited, HR was decreased in OS, PFS and CCS as the Apo A1 level was increasing.

### Association of Serum Apo A1 with Clinicopathological Characteristics and Patient Survival

After dichotomized our cohort into high Apo A1 subgroup and low Apo A1 subgroup, we first correlated the clinical characteristics to Apo A1 level as shown in Table 1. The Apo A1 level was correlated with gender (*P*=0.008), ISS stage (*P*<0.001), β2MG (*P*<0.001), albumin (*P*<0.001), hemoglobin (*P*<0.001), and SCr (*P*=0.015). Also, we compared the OS, PFS and CSS between lower Apo A1 group and higher Apo A1 group with Kaplan-Meier survival analysis (Figure 2D-2F). The median OS, PFS, and CCS in lower Apo A1 group and higher Apo A1 group were: 30 months vs. 64 months, 22 months vs. 33 months, and 31 months vs. 84 months respectively. The patients with higher Apo A1 showed longer OS rate (*P*<0.001), and the PFS and CSS showed consistent results (*P*<0.001 and *P*<0.001).

### Univariate and Multivariate Cox Proportional Hazard Analysis

In order to assess whether Apo A1 level was an independent prognostic factor, we performed the univariate and multivariate analysis of OS, PFS, and CSS. Other parameters available in our database also underwent univariate analysis. Clinical factors identified as statistically significant by univariate Cox regression analysis (Table S2) were included in the multivariate analysis (Table 2). Apo A1 level was identified as an independent prognostic factor in OS, PFS and CSS (*P*=0.020, *P*=0.028, and *P*=0.015 respectively), so was LDH (*P*<0.001, *P*=0.001 and *P*<0.001 respectively). β2MG was demonstrated as an independent factor only in OS and PFS (*P*=0.040 and *P*=0.014 respectively).

### Construction of a Prognostic Model for MM with Lasso Regression

**Figure 3:**
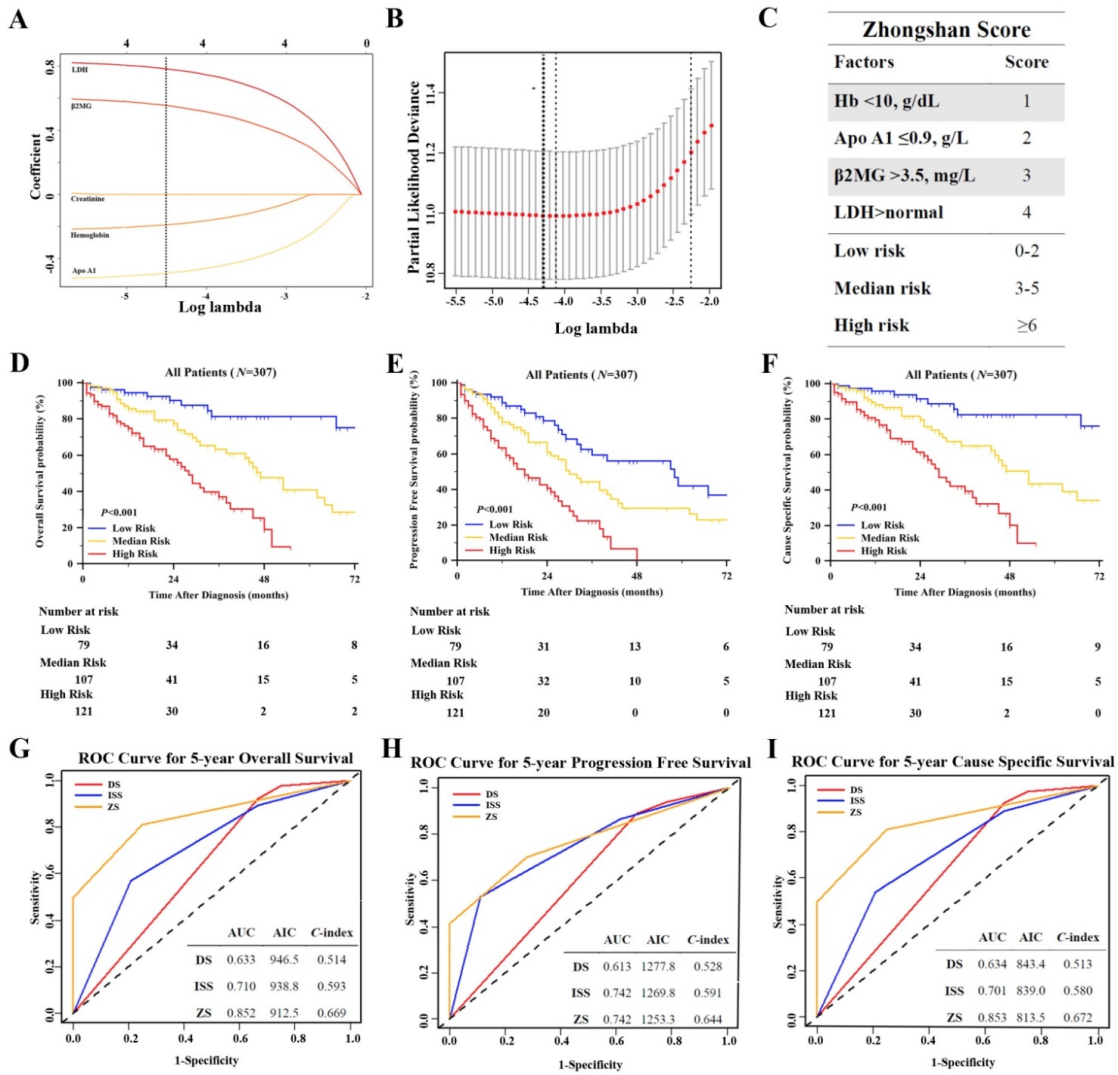
** Construction of a New Prognostic Model Zhongshan Score Based on Lasso Cox Regression and Evaluation of the Model.** [A], Lasso Cox regression of the 5 selected parameters. A dashed vertical line was drawn at the value when λ=0.014; [B], Partial likelihood deviance for the Lasso regression. The light dashed vertical line stood for the minimum partial likelihood deviance. The bold dashed vertical line stood for the partial likelihood deviance when λ=0.014; [C], The Zhongshan Score (ZS) with detailed parameters, points and stratification. Kaplan Meier survival analysis of ZS risk stratification of OS [D], PFS [E] and CSS [F] showed significant difference. ROC curves and *C*- index analysis of ZS, ISS, and DS for OS [G], PFS [H] and CSS [I]. The value of AUC, *C*- index and AIC were included in the figure.

We used Lasso Cox regression to select potential prognostic markers for OS, based on the result of univariate and multivariate analysis, and five parameters: Apo A1, SCr, hemoglobin, β2MG and LDH were included in the Lasso Cox regression model to determine the ideal coefficient for each potential prognostic marker. With the Lasso model, the coefficients for each factor were calculated when log(λ)=-4.3 and λ=0.014, with a partial likelihood deviance of 11.0 (Figure 3A-B). The coefficients for each parameter were as following: 0.42 for Apo A1<0.9g/L, 0.15 for hemoglobin<10g/dL, 0.57 for β2MG>3.5mg/L, and 0.82 for LDH above normal. According to the coefficients, a scoring index was derived from this model, named as Zhongshan Score, as shown in Figure 3C. The highest score was 10 points and the lowest was 0 points. We defined that the range of 0 to 2 points were considered as low risk, the range of 3 to 5 points were considered median risk, and total points over 6 were regarded as high risk. The OS, PFS and CSS survival curves were shown in Figure 3D-3F. Patients in high risk groups showed significantly worse OS when compared with other groups, with the median survival time as 87, 47, 28 months in the low, median and high risk groups respectively (*P*<0.001). The survival curve of PFS and CSS in these three groups showed consistent results (*P*<0.001, and *P*<0.001 respectively).

We compared the new model with the ISS and DS staging system with time dependent estimating ROC curve, *C*-index and AIC to evaluate the prognostic accuracy (Figure 3G-3I). The estimated AUC of five-year OS of Zhongshan Score showed statistical significance when compared with ISS and DS staging system (*P*=0.009 and *P*<0.001 respectively). The detailed results of AUC were listed in Table S3. The Zhongshan Score also showed higher *C*-index and lower AIC in OS, PFS, and CSS analysis, which might be indicating higher accuracy in predicting the outcomes than those with DS system and ISS system.

## Discussion

Few studies had focused on the relationship between lipid metabolism and prognosis of MM. In this study, we analyzed the pan lipid profile in different ISS stages, and identified that Apo A1 was an independent prognostic factor through both univariate and multivariate analysis. A higher Apo A1 level could imply longer OS, PFS and CCS. After that, we incorporated several parameters that have been identified by previous prognostic studies 3, 4, and constructed a new model called Zhongshan Score. This new system included four parameters: Apo A1, hemoglobin, β2MG and LDH. According to the scoring system, patients were classified into 3 risk groups, with a significant difference in OS, PFS and CSS. We also tested the model with time dependent ROC curve and *C*-index, of which the results indicated that Zhongshan Score had improved prognostic value when compared with other staging systems in our analysis. The Zhongshan Score had several advantages that the parameters were more easily available, and observer- independent.

The following question emerged after we came to the result of the analysis as above: why are Apo A1 capable of predicting the outcome of MM patients? Apo A1, a major protein component of HDL, has long been identified as an important part in lipid metabolism to bring lipid back to liver, and thus it has been regarded to have cardiovascular protective effect 23. In recent years several studies have indicated that Apo A1 can do more than cardiovascular protection. In a prospective investigation, the concentrations of HDL and Apo A1 were inversely associated with the risk of colon cancer 17. Such correlation has also been reported in lung cancer, breast cancer and nasopharyngeal cancer 24. A proteomic analysis has identified 22 proteins in myeloma patients that have a different level of expression from healthy controls, among which Apo A1 was found down regulated in myeloma patients 25, while the exact mechanism under these phenomenon was still unknown. An experiment by Maryam et al. has indicated that Apo A1 might have potent anti-tumorigenic effects on melanoma cells through several different mechanisms: increase CD8 T cell expression, increase anti-tumor macrophages, decrease angiogenesis, inhibit tumor growth, and reduce tumor invasion and metastasis 15. Furthermore, the anti-tumorigenic effect of Apo A1 could be associated with ATP-binding cassette transporter A1 (ABCA1) and ATP binding cassette transporter G1 (ABCG1), since Apo A1 is an acceptor of cholesterol at the first step of ABCA1/ABCG1-mediated HDL generation 26. Several studies have mentioned that, ABCA1, a major cellular cholesterol efflux transporter, might be involved in the pathogenesis of cancer 27. One study pointed out that ABCA1 down regulation was evident in prostate cancer due to promoter hypermethylation, leading to high intracellular cholesterol levels and an environment conductive to tumor progression 28. However, some studies revealed that ABCA1/ABCG1 had different role in tumor progression involving macrophage polarization 29. One study demonstrated that the absence of ABCG1 inhibited tumor growth through phenotypic shift of the macrophages from a tumor-promoting M2 to a tumor-fighting M1 within the tumor 30. In the mouse model of ovarian cancer and breast cancer model, Apo A1 mimetic peptide significantly inhibited the development of tumors 31, 32. However, no study of relation between Apo A1 and multiple myeloma was done. From these studies, we hypothesize that increasing serum Apo A1 level could also be a potential strategy for myeloma treatment. In conclusion, Apo A1 has several roles on different aspects of tumor progression and might inhibit tumor growth by downstream ABCA1/ABCG1 activation. Further studies are needed to discover the underlying mechanism of Apo A1-ABCA1/ABCG1-HDL axis on tumor progression.

Still no study had explored the role of Apo A1 in MM and thus further studies are needed to clarify the function of Apo A1 in myeloma. Besides, Apo A1 has a serum residence time of 4 days 33, and it could be influenced by several host characteristics, such as gender, nutrition status, liver metabolic function, insulin resistance, and previous medication history at the time of diagnosis 34. Apart from the potential anti-tumor effect, it could be a reflection of the overall health status of the patient.

Limitations of our study include the small number of cases and the single center database, which has made the survival curves unstable. The Zhongshan Score model needs to be validated by a multi-center investigation with a larger number of patients. We also had limited access to data on some prognostic markers such as FLC and cytogenetic analysis, which were presented in the article but not included in analysis due to poor data integrity. Further studies should be done to improve and validate the new prognostic model.

In conclusion, this is the first study identifying the prognostic value of whole lipid profile in MM. Apo A1, a novel prognosis indicator, was incorporated into Zhongshan Score, to further predict the clinical outcome of patients with MM. The findings have important implications for personalized follow-up after chemotherapy regimens and deeper understanding of the metabolic pathophysiology of MM.

## Supplementary Material

Supplementary document and tables.

## Figures and Tables

**Table 1:** Correlation between Serum Apo A1 Level and Patient Characteristics

Variables	Apo A1≤0.9g/L (%)	Apo A1>0.9g/L (%)	P
**Age**			0.924
≤65	75 (36.6)	130 (63.4)	
>65	36 (35.3)	66 (64.7)	
**Gender**			**0.008**
Female	30 (26.3)	84 (73.7)	
Male	81 (42.0)	112 (58.0)	
Isotype*			0.106
IgG	55 (36.2)	97 (63.8)	
IgA	37 (43.5)	48 (56.5)	
IgD	4 (50.0)	4 (50.0)	
Light chain	11 (22.4)	38 (77.6)	
No secretary	3 (25.0)	9 (75.0)	
**ISS Stage**			**<0.001**
I	10 (16.9)	49 (83.1)	
II	32 (31.7)	69 (68.3)	
III	69 (46.9)	78 (53.1)	
**Albumin, g/dL**			**<0.001**
<3.5	85 (45.9)	100 (54.1)	
≥3.5	26 (21.3)	96 (78.7)	
**β2-MG, mg/L**			**<0.001**
<3.5	19 (20.0)	76 (80.0)	
≥3.5	92 (43.4)	120 (56.6)	
**LDH, mmol/L**			0.916
Normal	83 (35.8)	149 (64.2)	
High	28 (37.3)	47 (62.7)	
**Hemoglobin, g/dL**			**<0.001**
<10	87 (50.0)	87 (50.0)	
≥10	24 (17.6)	109 (82.4)	
**SCr, mg/dL**			**0.019**
<2	81 (32.8)	166 (67.2)	
≥2	30 (50.0)	30 (50.0)	
**Ca, mg/dL**			
<10	81 (33.9)	158 (66.1)	0.160
≥10	30 (44.1)	38 (55.9)	

Abbreviation: ISS, International Staging System; Apo A1, apolipoprotein A1; LDH, lactate dehydrogenase; β2MG, β2 microglobulin; SCr, serum creatinine. *Isotype: only one case in biclonal isotype, as seen in supplement materials

**Table 2:** Multivariate Cox Regression Analysis of Overall Survival, Progression Free Survival and cause specific survival

Variables	OS			PFS			CSS	
	HR(95%CI)	*P*		HR(95%CI)	*P*		HR(95%CI)	*P*
**Apo A1, g/l**								
≤0.9	Reference			Reference			Reference	
>0.9	0.609(0.403-0.922)	**0.020**		0.668(0.467-0.956)	**0.028**		0.585(0.381-0.899)	**0.015**
**β2-MG, mg/l**								
<3.5	Reference			Reference			Reference	
≥3.5	1.771(1.030-3.047)	**0.040**		1.704(1.118-2.600)	**0.014**		1.637(0.933-2.871)	0.087
**LDH**								
Normal	Reference			Reference			Reference	
High	2.385(1.509-3.769)	**<0.001**		1.938(1.306-2.875)	**0.001**		2.551(1.604-4.057)	**<0.001**
**Scr, mg/dl**								
<2	Reference						Reference	
≥2	0.955(0.572-1.595)	0.860					1.098(0.652-1.849)	0.726
**Hb, g/dl**								
<10	Reference			Reference			Reference	
≥10	0.774(0.484-1.236)	0.286		0.946(0.641-1.396)	0.779		0.741(0.451-1.216)	0.238
**Isotype**								
IgG	Reference							
IgA	1.464(0.914-2.345)	0.114						
IgD	1.308(0.461-3.717)	0.616						
Light chain	1.659(0.932-2.853)	0.087						
Non-secretary	2.360(0.709-7.850)	0.164						

## Abbreviations

MM: multiple myeloma
Apo A1: apolipoprotein A1
OS: overall survival
PFS: progression free survival
CSS: cause specific survival
β2M: β2-microglobulin
LDH: lactate dehydrogenase
SCr: serum creatinine
ISS: International Staging System
DS: Durie and Salmon system

## References

[1] Kumar SK, Dispenzieri A, Lacy MQ, Gertz MA, Buadi FK, Pandey S (2014). Continued improvement in survival in multiple myeloma: changes in early mortality and outcomes in older patients. Leukemia.

[2] Röllig C, Knop S, Bornhäuser M (2015). Multiple myeloma. The Lancet.

[3] Durie BG, Salmon SE (1975). A clinical staging system for multiple myeloma. Correlation of measured myeloma cell mass with presenting clinical features, response to treatment, and survival. Cancer.

[4] Greipp PR, San Miguel J, Durie BG, Crowley JJ, Barlogie B, Blade J (2005). International staging system for multiple myeloma. Journal of clinical oncology: official journal of the American Society of Clinical Oncology.

[5] Palumbo A, Avet-Loiseau H, Oliva S, Lokhorst HM, Goldschmidt H, Rosinol L (2015). Revised International Staging System for Multiple Myeloma: A Report From International Myeloma Working Group. Journal of clinical oncology: official journal of the American Society of Clinical Oncology.

[6] Liu Q, Luo Q, Halim A, Song G (2017). Targeting lipid metabolism of cancer cells: A promising therapeutic strategy for cancer. Cancer letters.

[7] Yavasoglu I, Tombuloglu M, Kadikoylu G, Donmez A, Cagirgan S, Bolaman Z (2008). Cholesterol levels in patients with multiple myeloma. Annals of hematology.

[8] Hachem H, Favre G, Raynal G, Soula G (1983). [Plasma lipoproteins and multiple myeloma. Variations of lipid constituents of HDL and apolipoproteins A1 and B]. Annales de biologie clinique.

[9] Dmoszynska A, Podhorecka M, Rolinski J, Soroka-Wojtaszko M (2004). Influence of lovastatin on BCL-2 and BAX expression by plasma cells and T lymphocytes in short-term cultures of multiple myeloma bone marrow mononuclear cells. Polish journal of pharmacology.

[10] Slawinska-Brych A, Zdzisinska B, Mizerska-Dudka M, Kandefer-Szerszen M (2013). Induction of apoptosis in multiple myeloma cells by a statin-thalidomide combination can be enhanced by p38 MAPK inhibition. Leukemia research.

[11] Hus M, Grzasko N, Szostek M, Pluta A, Helbig G, Woszczyk D (2011). Thalidomide, dexamethasone and lovastatin with autologous stem cell transplantation as a salvage immunomodulatory therapy in patients with relapsed and refractory multiple myeloma. Annals of hematology.

[12] Sanfilippo KM, Keller J, Gage BF, Luo S, Wang TF, Moskowitz G (2016). Statins Are Associated With Reduced Mortality in Multiple Myeloma. Journal of clinical oncology: official journal of the American Society of Clinical Oncology.

[13] Schmidmaier R, Baumann P, Bumeder I, Meinhardt G, Straka C, Emmerich B (2007). First clinical experience with simvastatin to overcome drug resistance in refractory multiple myeloma. European journal of haematology.

[14] Schmidmaier R, Baumann P, Simsek M, Dayyani F, Emmerich B, Meinhardt G (2004). The HMG-CoA reductase inhibitor simvastatin overcomes cell adhesion-mediated drug resistance in multiple myeloma by geranylgeranylation of Rho protein and activation of Rho kinase. Blood.

[15] Zamanian-Daryoush M, Lindner D, Tallant TC, Wang Z, Buffa J, Klipfell E (2013). The cardioprotective protein apolipoprotein A1 promotes potent anti-tumorigenic effects. The Journal of biological chemistry.

[16] Borgquist S, Butt T, Almgren P, Shiffman D, Stocks T, Orho-Melander M (2016). Apolipoproteins, lipids and risk of cancer. International journal of cancer.

[17] van Duijnhoven FJ, Bueno-De-Mesquita HB, Calligaro M, Jenab M, Pischon T, Jansen EH (2011). Blood lipid and lipoprotein concentrations and colorectal cancer risk in the European Prospective Investigation into Cancer and Nutrition. Gut.

[18] Jiang R, Yang ZH, Luo DH, Guo L, Sun R, Chen QY (2014). Elevated apolipoprotein A-I levels are associated with favorable prognosis in metastatic nasopharyngeal carcinoma. Medical oncology.

[19] Shi J, Yang H, Duan X, Li L, Sun L, Li Q (2016). Apolipoproteins as Differentiating and Predictive Markers for Assessing Clinical Outcomes in Patients with Small Cell Lung Cancer. Yonsei medical journal.

[20] Hariprasad G, Hariprasad R, Kumar L, Srinivasan A, Kola S, Kaushik A (2013). Apolipoprotein A1 as a potential biomarker in the ascitic fluid for the differentiation of advanced ovarian cancers. Biomarkers: biochemical indicators of exposure, response, and susceptibility to chemicals.

[21] Rajkumar SV, Dimopoulos MA, Palumbo A, Blade J, Merlini G, Mateos MV (2014). International Myeloma Working Group updated criteria for the diagnosis of multiple myeloma. The Lancet Oncology.

[22] Kumar S, Paiva B, Anderson KC, Durie B, Landgren O, Moreau P (2016). International Myeloma Working Group consensus criteria for response and minimal residual disease assessment in multiple myeloma. The Lancet Oncology.

[23] Boekholdt SM, Arsenault BJ, Hovingh GK, Mora S, Pedersen TR, Larosa JC (2013). Levels and changes of HDL cholesterol and apolipoprotein A-I in relation to risk of cardiovascular events among statin-treated patients: a meta-analysis. Circulation.

[24] Hamrita B, Ben Nasr H, Gabbouj S, Bouaouina N, Chouchane L, Chahed K (2011). Apolipoprotein A1 -75 G/A and +83 C/T polymorphisms: susceptibility and prognostic implications in breast cancer. Molecular biology reports.

[25] Zhang HT, Tian EB, Chen YL, Deng HT, Wang QT (2015). Proteomic analysis for finding serum pathogenic factors and potential biomarkers in multiple myeloma. Chinese medical journal.

[26] Smith B, Land H (2012). Anticancer activity of the cholesterol exporter ABCA1 gene. Cell reports.

[27] Ding X, Zhang W, Li S, Yang H (2019). The role of cholesterol metabolism in cancer. American Journal of Cancer Research.

[28] Lee BH, Taylor MG, Robinet P, Smith JD, Schweitzer J, Sehayek E (2013). Dysregulation of cholesterol homeostasis in human prostate cancer through loss of ABCA1. Cancer research.

[29] Yvan-Charvet L, Wang N, Tall AR (2010). Role of HDL, ABCA1, and ABCG1 transporters in cholesterol efflux and immune responses. Arteriosclerosis, thrombosis, and vascular biology.

[30] Sag D, Cekic C, Wu R, Linden J, Hedrick CC (2015). The cholesterol transporter ABCG1 links cholesterol homeostasis and tumour immunity. Nature communications.

[31] Su F, Kozak KR, Imaizumi S, Gao F, Amneus MW, Grijalva V (2010). Apolipoprotein A-I (apoA-I) and apoA-I mimetic peptides inhibit tumor development in a mouse model of ovarian cancer. Proceedings of the National Academy of Sciences of the United States of America.

[32] Cedo L, Garcia-Leon A, Baila-Rueda L, Santos D, Grijalva V, Martinez-Cignoni MR (2016). ApoA-I mimetic administration, but not increased apoA-I-containing HDL, inhibits tumour growth in a mouse model of inherited breast cancer. Scientific reports.

[33] Schaefer EJ, Zech LA, Jenkins LL, Bronzert TJ, Rubalcaba EA, Lindgren FT (1982). Human apolipoprotein A-I and A-II metabolism. Journal of lipid research.

[34] Pont F, Duvillard L, Florentin E, Gambert P, Verges B (2002). High-density lipoprotein apolipoprotein A-I kinetics in obese insulin resistant patients. An in vivo stable isotope study. International journal of obesity and related metabolic disorders: journal of the International Association for the Study of Obesity.

