# Supplementary material for: Identification of High Serum Apolipoprotein A1 as a Favorable Prognostic Indicator in Patients with Multiple Myeloma
Source: J Cancer. 2019 Aug 27;10(20):4852–9. doi: 10.7150/jca.31357 (PMC6775509; doi:10.7150/jca.31357)
Supplement: Supplementary file 1 — Supplementary document and tables. [file jcav10p4852s1.pdf]

Table S1. Characteristics of Patients with Multiple Myeloma

Variables	No. of Patients (%) (*N*= 307)	Variables	No. of Patients (%) (*N*= 307)
**Age, year**		**Albumin, g/dL**	
≤ 65	205 (66.8)	<3.5	185 (60.3)
>65	102 (33.2)	≥3.5	122 (39.7)
**Gender**		**β2-MG, mg/L**	
Female	114 (37.1)	<3.5	95 (30.9)
Male	193 (62.9)	≥3.5	212 (69.1)
**Isotype**		**FLC ratio***	
IgG	152 (49.5)	Normal	73 (59.3)
IgA	85 (27.7)	Abnormal	50 (40.7)
IgD	8 (2.6)	**Lipid profile, median (mean±SD)**	
Light chain	49 (16.0)	Cholesterol, mmol/L	3.37 (3.44±1.26)
Biclonal	1 (0.3)	Triglyceride, mmol/L	1.20 (1.45±1.18)
Non-Secretory	12 (3.9)	LDL, mmol/L	1.76 (1.88±0.98)
**ISS Stage**		HDL, mmol/L	0.88 (0.93±0.34)
I	59 (19.2)	Apo A1, g/L	1.02 (1.04±0.31)
II	101 (32.9)	Apo B, g/L	0.64 (0.65±0.28)
III	147 (47.9)	**Cytogenetic Abnormality***	
**LDH, U/L**		Del(17p)	38 (39.6)
Normal	232 (75.6)	Del(13q)	53 (55.2)
High	75 (24.4)	1q21 amplification	54 (56.3)
**Hb, g/dL**		IgH translocation	46 (47.9)
<10	174 (56.7)	**Initial Treatment**	
≥10	133 (43.3)	PIs involved	171* (55.7)
**SCr, mg/dL**		IMiDs involved	162* (52.8)
<2	247 (80.5)	No new drugs	38 (12.4)
≥2	60 (19.5)	No data	12 (3.9)
**Ca, mg/dL**			
<10	239(77.9)		
≥10	68(22.1)		

Abbreviation: ISS, International Staging System; LDH, lactate dehydrogenase; SCr, serum creatinine; β2MG, β2 microglobin; FLC, free light chain; SD, standard deviation; LDL, low density lipoprotein; HDL, high density lipoprotein, Apo A1, apolipoprotein A1; Apo B, apolipoprotein B; PI, protease inhibitor; IMiDs, immunomodulatory drugs;

*Records of FLC were available in 123 patients.

*fluorescence in situ hybridization (FISH) test results were available in 96 patients.

*76 patients received both IMiDs and Bortezomib-based treatment were counted twice.

**Table S2. Univariate Cox Regression of Dichotomized Patient Characteristics**						
**Variables**	OS<br>HR (95%CI)	*P*	PFS<br>HR (95%CI)	*P*	CSS<br>HR (95%CI)	*P*
**Age**		0.119		0.305		0.147
≤ 65	Reference		Reference		Reference	
>65	1.384 (0.922-2.077)		1.207 (0.844-1.726)		1.376 (0.896-2.113)	
**Gender**		0.419		0.577		0.628
Male	Reference		Reference		Reference	
Female	0.839 (0.550-1.282)		0.903 (0.631-1.291)		0.896 (0.576-1.394)	
**Isotype***						
IgG	Reference		Reference		Reference	
IgA	1.667 (1.051-2.646)	**0.031**	1.425 (0.968-2.099)	0.074	1.453 (0.893-2.363)	0.134
IgD	1.565 (0.560-4.375)	0.395	0.833 (0.303-2.285)	0.724	1.576 (0.563-4.412)	0.389
Light chain	1.996 (1.163-3.425)	**0.013**	1.403 (0.861-2.284)	0.176	1.653 (0.921-2.968)	0.094
No secretary	1.305 (0.403-4.228)	0.659	0.653 (0.206-2.075)	0.472	1.360 (0.419-4.414)	0.611
**Apo A1, g/L**		**<0.001**		**0.002**		**<0.001**
≤0.9	Reference		Reference		Reference	
>0.9	0.506 (0.342-0.749)		0.578 (0.411-0.814)		0.469 (0.310-0.708)	
**Albumin, g/dL**		0.884		0.180		0.571
<3.5	Reference		Reference		Reference	
≥3.5	0.970 (0.650-1.449)		0.785 (0.552-1.117)		0.883 (0.576-1.354)	
**β2MG, mg/L**		**<0.001**		**<0.001**		**<0.001**
<3.5	Reference		Reference		Reference	
≥3.5	2.522 (1.563-4.069)		2.034 (1.383-2.989)		2.360 (1.441-3.869)	
**LDH**		**<0.001**		**<0.001**		**<0.001**
Normal	Reference		Reference		Reference	
High	2.979 (1.931-4.595)		2.210 (1.500-3.256)		3.136 (1.987-4.948)	
**Hb, g/dL**		**0.002**		**0.015**		**0.001**
<10	Reference		Reference		Reference	
≥10	0.522 (0.344-0.793)		0.649 (0.459-0.919)		0.479 (0.307-0.747)	
**SCr, mg/dL**		**0.037**		0.083		**0.033**
<2	Reference		Reference		Reference	
≥2	1.661 (1.035-2.665)		1.463 (0.954-2.243)		1.720 (1.046-2.827)	
**Ca, mg/dL**		0.110		0.178		0.149
<10	Reference		Reference		Reference	
≥10	1.438 (0.923-2.240)		1.312 (0.885-1.945)		1.415 (0.885-2.263)	

Abbreviation: ISS, International Staging System; Apo A1, apolipoprotein A1; LDH, lactate dehydrogenase; β2MG, β2 microglobulin; SCr, serum creatinine.

Table S3. Evaluation of the Three Prognostic Model					
		**AUC**	**AIC**	**BIC**	***C*-index**
**OS**	**DS**	0.633 (0.533-0.734)	946.5	950.2	0.514
	**ISS**	0.710 (0.603-0.819)	938.8	942.5	0.593
	**ZS**	0.852 (0.788-0.917)	912.5	916.2	0.669
**PFS**	**DS**	0.613 (0.497-0.846)	1277.8	1281.5	0.528
	**ISS**	0.742 (0.638-0.846)	1269.8	1273.6	0.591
	**ZS**	0.742 (0.654-0.830)	1253.3	1257	0.644
**CSS**	**DS**	0.634 (0.534-0.734)	843.4	847.1	0.513
	**ISS**	0.701 (0.590-0.813)	839	842.7	0.58
	**ZS**	0.853 (0.786-0.919)	813.5	817.2	0.672

Abbreviation: DS, Durie and Salmon system; ISS, International Staging System; ZS, Zhongshan Score;

Supplemental document 1

The International Myeloma Working Group (IMWG) consensus criteria for response and minimal residual disease assessment in multiple myeloma, progressive disease is defined as following:

Any one or more of the following:

- Increase of 25% from lowest confirmed response value in one or more of the following criteria:
  - ✓ Serum M-protein (absolute increase must be ≥0.5 g/dL);
  - ✓ Serum M-protein increase ≥1 g/dL, if the lowest M component was ≥5 g/dL;
  - ✓ Urine M-protein (absolute increase must be ≥200 mg/24 hours);
  - ✓ In patients without measurable serum and urine M-protein levels, the difference between involved and uninvolved FLC levels (absolute increase must be >10 mg/dL);
  - ✓ In patients without measurable serum and urine M-protein levels and without measurable involved FLC levels, bone marrow plasma-cell percentage irrespective of baseline status (absolute increase must be ≥10%);
- Appearance of a new lesion(s), ≥50% increase from nadir in SPD** of >1 lesion, or ≥50% increase in the longest diameter of a previous lesion >1 cm in short axis;
- ≥50% increase in circulating plasma cells (minimum of 200 cells per microL) if this is the only measure of disease.
